# Translation and validation of the Swedish version of the EORTC LMC-21, the disease-specific questionnaire for assessing health-related quality of life in patients with colorectal liver metastases

**DOI:** 10.1186/s12876-025-03835-w

**Published:** 2025-04-22

**Authors:** Anna Lindhoff Larsson, Peter Holka, Bengt Isaksson, Oskar Hemmingsson, Per Sandström, Bergthór Björnsson

**Affiliations:** 1 Department of Surgery in Linköping, and Department of Biomedical and Clinical Sciences, Linköping University, Linköping, Sweden; 2 Department of Surgery, Clinical Sciences Lund, Lund University and Skåne University Hospital, Lund, Sweden; 3 Department of Surgical Sciences, Sweden Department of Surgery, Uppsala University, Akademiska Hospital, Uppsala, Sweden; 4 Department of Surgical and Perioperative Sciences, Surgery, Umeå University, Umea, Sweden

**Keywords:** Quality of life (QoL), Colorectal cancer, EORTC QLQ-LMC21, Psychometric validation

## Abstract

**Purpose:**

The aim of this study was to translate the health-related quality-of-life questionnaire EORTC QLQ-LMC21 into Swedish and to test its clinical and psychometric reliability and validity in patients with liver metastases from colorectal cancer (CRC) undergoing surgical treatment.

**Methods:**

The Swedish versions of the EORTC QLQ-C30 and EORTC QLQ-LMC21 were administered to 250 patients with liver metastases from CRC in four Swedish hospitals before and 3 months after surgical treatment. Psychometric validation of the questionnaire´s structure, reliability, and convergent and divergent validity was performed.

**Results:**

Data from 242 (97%) patients were suitable for analysis. The QLQ-LMC21 was found to be sensitive to changes over time. Cronbach´s alpha coefficient indicated good internal consistency, ranging from 0.84 to 0.89. Test–retest reliability was evaluated in 120 patients (49%), and the intraclass correlation coefficient (ICC) indicated good reproducibility, ranging from 0.67 to 0.93. Convergent and discriminant validity were demonstrated adequately in the multitrait scaling analysis. There were weak correlations between the QLQ-C30 and QLQ-LMC21, which confirms that the health problems addressed by the QLQ-LMC21 are different from those addressed by the QLQ-C30.

**Conclusions:**

The Swedish version of the EORTC QLQ-LMC21 proved to be a valid and reliable questionnaire to use in conjunction with the EORTC QLQ-C30.

**Supplementary Information:**

The online version contains supplementary material available at 10.1186/s12876-025-03835-w.

## Introduction

Colorectal cancer (CRC) is the third most common type of cancer in the Western world and the fourth most common cause of cancer-related death worldwide [1]. In Sweden, approximately 6700 patients are diagnosed with CRC annually, and 15–20% of these patients are also diagnosed with synchronous liver metastases. The main cause of death is dissemination of the disease. The liver is one of the most common sites of metastasis and is the leading cause of cancer-related death [2]. Surgical or ablative treatment for liver metastases are the only potentially curative treatments for these patients, with a reported 5-year survival of 25 to 60% for patients with metachronous liver metastases; for patients with synchronous disease, the 5-year survival is reported to be 25–40% [1, 3].

The rapid development of new technologies in medicine and surgery makes it possible to improve methods of treating certain diseases. Surgery has become more aggressive to allow for resection of more liver metastases and thus being able to offer treatment with curative intention to more patients. Whether the patient has a good quality of life after surgery and treatment is still debated. There is a growing interest in assessing how disease and treatment affect patients' lives. A common measurement instrument for evaluating quality of life in cancer patients is the European Organization for Research and Treatment of Cancer Core Quality of Life Questionnaire (EORTC QLQ-C30). The questionnaire was developed by the EORTC Quality of Life Group to measure general quality of life in cancer patients. It has been validated through numerous studies and is available in more than 100 languages [4, 5]. The questionnaire consists of 30 questions that assess function (physical, role, emotional, social and cognitive), symptoms (fatigue, nausea and vomiting, dyspnoea, insomnia, anorexia, constipation, diarrhoea and pain) and overall health status. Disease-specific questionnaires are linked to this questionnaire*.* The EORTC QLQ-LMC21 is a questionnaire developed and validated by the EORTC Quality of Life Group for patients with liver metastases from CRC at varying stages of disease and treatment [6, 7]. The questionnaire consists of 21 disease-specific questions. Questionnaires previously developed in another language must be validated in a systematic way before their reliability can be tested. However, the Swedish version has not yet been translated and validated.

The aim of this study was to translate the health-related quality-of-life questionnaire EORTC QLQ-LMC21 into Swedish and to test its clinical and psychometric reliability and validity in patients with liver metastases from CRC undergoing surgical treatment.

## Materials and methods

### Translation and procedures

Starting in 2015, the QLQ-LMC21 was translated into Swedish in collaboration with the EORTC Quality of Life Group according to the instructions in the Translation Procedure Manual [8].

The translation coordinator ascertained whether parts of the LMC21 (instructions, questionnaire items, response categories) were available in the Swedish language. The existing translations of these sections were used.

The first translation from English into Swedish was performed by two independent native Swedish-speaking authorized translators with good knowledge of the English language and extensive experience in translating medical documents.

The two versions of the translation were compared and discussed by the two translators and project manager. The conclusion of that debate was then discussed with a group consisting of two physicians and one professor who has supervised numerous quality-of-life studies. The project manager coordinated the discussion. The first version of the Swedish translation was based on the conclusion from these discussions.

The first version of the translation was then back-translated to English by two native English speakers with a good command of the Swedish language (one authorized translator and one oncology nurse with research experience in the quality-of-life questionnaire).

The Swedish version of the questionnaire was based on a comparison of the two translations (English into Swedish and Swedish into English) and the original questionnaire.

The 21 questions in the QLQ-LMC21 were pilot tested in December 2016 with 12 patients, 5 women and 7 men. Informed consent was obtained from all study participants. The mean age was 68 years (range 36–82 years), and all patients were native Swedish speakers diagnosed with liver metastases from CRC. Five patients with rectal cancer and seven with colonic cancer were diagnosed between 2007 and 2016. Eight patients had previously undergone intestinal surgery. Eleven patients were waiting for surgery for liver metastases; six of them were receiving ongoing oncological treatment. One patient recently underwent surgery for liver metastasis. Five patients were retired, and the remaining patients were professionals, including 2 warehouse workers, 1 entrepreneur, 1 plumber, 1 consultant, and 1 business manager. All patients were asked to complete the QLQ-C30 and QLQ-LMC21 and subsequent questions for this pilot test at home and return it by post. Nine follow-up interviews were performed in the hospital, and 3 were performed by telephone.

The proposed changes were discussed with the EORTC Quality of Life Group experts, and then the final Swedish version was adopted.

In accordance with the EORTC Quality of Life Group's guidelines for the development of quality-of-life instruments, a minimum of 10 patients per question is needed. The questionnaire consists of 21 questions, so a total of 210 patients was necessary for validation of the instrument. The study planned for 250 patients to address possible dropouts.

For the validation of the final version of the QLQ-LMC21, 250 consecutive patients were recruited from four university hospitals in Sweden between May 2017 and October 2021. Informed consent was obtained from all study participants.

The inclusion criteria were as follows: patients over 18 years of age who had been diagnosed with CRC and liver metastases with an expected survival of more than 4 weeks.

The exclusion criteria were as follows: other malignancies and inability to understand and complete the questionnaires.

All patients completed the EORTC QLQ-C30 and EORTC QLQ-LMC21 before and three months after surgery.

A validation study should involve a large heterogeneous group of patients and include patients who are representative of the full spectrum of intended patients. To ensure that the study group was a representative sample of the target group, the following data were collected:

- Sociodemographic data: Sex, age, marital status, education, employment.
- Clinical data: Tumour location/recurrence; tumour stage; pre-, peri- and postoperative treatment/palliative treatment; ECOG.

### Questionnaires

The EORTC QLQ-C30 consists of 30 health-related items that assess functioning (physical, role, emotional, social and cognitive), symptoms (fatigue, nausea and vomiting, dyspnoea, insomnia, anorexia, constipation, diarrhoea and pain) and general health status. The questionnaire involves five function scales, three symptom scales, six single items and a global health status. The Swedish version has previously been validated and found to be a reliable and valid measure of the quality of life of cancer patients in multicultural clinical research settings [9].

The EORTC QLQ-LMC21 includes 21 items developed to evaluate disease-specific symptoms in patients with liver metastases from CRC. The 21 questions are designed as four scales (nutrition, fatigue, pain and emotional problems) and nine single items (weight loss, problems with taste, dry mouth, sore mouth, peripheral neuropathy, jaundice, contact with friends, talking about feelings and sex life).

The response options in both questionnaires are rated on a Likert-type scale (not at all, a little, quite a lot and very much). The values obtained from the completed questionnaires were converted into scales from 0 to 100 in accordance with the EORTC Scoring Manual [10]. Higher values on the function scales and global health status represent better function and a better quality of life, while higher values on the symptom scales indicate more problems and symptoms, suggesting a worse quality of life.

The Swedish versions of the QLQ-C30 and QLQ-LMC21 modules and their scoring manuals were provided by the EORTC department at the request of the authors of this study.

### Ethics

This study was conducted in accordance with the 2013 Declaration of Helsinki and was approved by the Swedish Ethical Review Authority (Dnr: M119 - 09.)

### Statistical analysis

Sociodemographic and clinical variables of the participants in the validation stage were analysed using descriptive statistics and are presented as means and percentages.

To examine responsiveness to changes in health status over time, mean scores were calculated, and a paired t test was used on the QLQ-C30 and QLQ-LMC21 before and after hepatectomy.

To evaluate the stability and repeatability of the items in the QLQ-LMC21, the questionnaire was sent to 120 patients who would be eligible for a test–retest within 14 days before surgery. These data were used to calculate the intraclass correlation coefficients, and good correlation was defined as > 0.80 [11].

The construct validity of the QLQ-LMC21 was assessed using Spearman's nonparametric correlation coefficient. Discriminant validity was assessed by correlating each item with all scales, excluding the scale in which the specific item was included. To evaluate convergent validity, each item was correlated with its own scale. In this study, convergent validity was established if the correlation between an item and its own scale was 0.40 or higher (corrected for overlap) [11]. The internal consistency of the QLQ-LMC21 was calculated using Cronbach’s alpha. Values above 0.7 were considered acceptable, values above 0.8 were considered good and values above 0.9 were considered excellent [11].

To establish concurrent validity in this study, the comparable subscales of the QLQ-C30 and the QLQ-LMC21 were analysed with Spearman's correlation coefficient.

All analyses were performed using IBM SPSS (version 24; IBM Corp, Armonk, NY, USA).

## Results

No patients in the pilot test experienced any difficulties in completing the questionnaires. None of the questions were upsetting or contained difficult words. The patients reported that it took approximately 15–25 min to complete the questionnaire, and all patients completed the questionnaire without assistance. Five patients provided comments on the questionnaire. One patient thought it should be clarified that the items refer to the most recent week. One patient thought that questions 37 and 43 were very similar, one patient mentioned that questions 39 and 40 were similar, and a third patient thought that questions 43 and 44 were similar. One person commented on question number 50 that not everyone has a family and perhaps the word “family” could be replaced with “relatives”. The questionnaire is available as Supplement 1.

The survey compliance of the 242 patients was 97% at baseline and 83% at the 3-month postoperative follow-up. Preoperatively, 3% of item responses were missing, and at follow-up, this increased to 18%. The majority of the missing items were from the question that assessed sexual function. The sociodemographic and clinical characteristics of the patients are presented in Table 1.

**Table 1:** Sociodemographic and clinical characteristics of patients

Variable	Value
Gender	
Male	152 (62.8)
Female	90 (37.2)
Age, mean years ± SD (range)	69 ± 9.7 (39 - 88)
Marital status	
Married/Cohabiting	168 (69.4)
Single	50 (20.7)
Widowed	4 (1.6)
Live separately	7 (2.9)
No information available	13 (5.4)
Education^a^	
Primary	76 (31.4)
High school, 2 y	43 (17.8)
High school, 3-4 y	34 (14.1)
University	63 (26.0)
No information available	26 (10.7)
Employment^a^	
Employed*	74 (30.6)
Retired	147 (60.7)
Unemployed	1 (0.4)
Sick leave	7 (2.9)
No information available	13 (5.4)
Primary colorectal surgery	
Hemicolectomy	80 (33.1)
Anterior resection	44 (18.2)
Abdominoperineal excision	32 (13.2)
Other/unknown	9 (3.7)
Liver first	73 (30.2)
No information available	4 (1.6)
Primary colorectal tumour, T-stage	
1	4 (1.6)
2	11 (4.6)
3	133 (54.9)
4	76 (31.4)
No information available	18 (7.4)
ECOG performance status	
0	141 (58.3)
1	76 (31.4)
2	18 (7.4)
3	0 (0)
4	0 (0)
No information available	7 (2.9)
Detection of liver metastases	
Synchronous with primary tumour	139 (57.4)
Routine follow up scan	93 (38.4)
Symptoms on follow up	6 (2.5)
Unknown	2 (0.8)
No information available	2 (0.8)
Treatment	
Hemihepatectomies	44 (18.2)
Single or multiple segmentectomies	85 (35.1)
Single or multiple Wedge resections	88 (36.4)
Palliative chemotherapy	3 (1.2)
Radiofrequency ablation	2 (0.8)
Other operation	9 (3.7)
Other	8 (3.3)
No information available	3 (1.2)

Values in parentheses are percentages

*Full time/part time/student

The QLQ-C30 and the QLQ-LMC21 were found to be sensitive to changes over time. In the QLQ-C30, patients reported significantly lower levels of physical function, role function, cognitive function, social function and global quality of life three months after hepatectomy. They also reported significantly higher levels of pain, fatigue and nausea/vomiting. The responses to the scales and items in the QLQ-LMC21 revealed significantly increased levels of nutritional problems, fatigue, pain, weight loss, sore mouth and peripheral neuropathy three months after surgery. The questionnaire also indicated that patients had increased social and sexual problems postoperatively (Table 2).

**Table 2:** Mean scores on QLQ-C30 and QLM-LM21 before and after hepatectomy

Variable	Hepatectomy (n= 202)		*P ‡*
	Before	After	
Functional scales, QLQ-C30*			
Physical function	86 (15)	80 (19)	<0.001
Role function	76 (26)	66 (33)	<0.001
Emotional function	81 (19)	80 (21)	0.470
Cognitive function	89 (15)	86 (19)	0.015
Social function	78 (24)	75 (25)	0.021
Global quality of life	71 (19)	65 (20)	<0.001
Symptom scales**			
Pain	11 (19)	19 (25)	<0.001
Fatigue	28 (21)	35 (24)	<0.001
Nausea and vomiting	4 (12)	7 (13)	0.004
Scales and items, QLQ-LMC21**			
Nutritional problems	11 (18)	16 (24)	<0.001
Fatigue	36 (26)	42 (29)	0.004
Pain	13 (17)	19 (21)	<0.001
Emotional problems	32 (22)	30 (24)	0.228
Weight loss	11 (19)	15 (25)	0.015
Taste problems	16 (26)	18 (28)	0.167
Dry mouth	21 (26)	22 (30)	0.808
Sore mouth	8 (17)	11 (24)	0.044
Peripheral neuropathy	26 (32)	33 (34)	0.002
Jaundice	1 (5)	0 (3)	0.319
Social problems	16 (28)	23 (31)	<0.001
Talking problems	10 (20)	17 (76)	0.202
Sexual problems	36 (39)	46 (39)	<0.001

Values are mean (SD)

*A higher score indicates better function

**A higher score indicates more symptoms

^‡^Paired t test

### Test–retest reliability

In the evaluation of stability and repeatability, the intraclass correlation coefficient (ICC) indicated good reproducibility, with a range from 0.67 to 0.93. Four items had scores lower than 0.80: Item 33 (weight loss), Item 39 (pain in the stomach), Item 40 (discomfort in the stomach) and Item 46 (talking about feelings) (Table 3).

**Table 3:** Test-retest reliability

	ICC* from the test-retest group (n = 120)
31. Have you had trouble with eating?	0.80
32. Have you felt full up too quickly after beginning to eat?	0.80
33. Have you worried about losing weight?	0.77
34. Have you had problems with your sense of taste?	0.92
35. Have you had a dry mouth?	0.93
36. Have you had a sore mouth or tongue?	0.86
37. Have you been less active than you would like to be?	0.87
38. Have you had tingling hands or feet?	0.85
39. Have you had pain in your stomach area?	0.70
40. Have you had discomfort in your stomach area?	0.67
41. Have your skin or eyes been yellow (jaundiced)?	0.89
42. Have you had pain in your back?	0.81
43. Have you felt slowed down?	0.81
44. Have you felt lacking in energy?	0.84
45. Have you had trouble having social contact with friends?	0.82
46. Have you had trouble talking about your feelings to your family or friends?	0.68
47. Have you felt stressed?	0.83
48. Have you felt less able to enjoy yourself?	0.86
49. Have you worried about your health in the future?	0.87
50. Were you worried about your family in the future?	0.88
51. Has the disease or treatment affected your sex life?	0.92

*ICC = Intraclass correlation coefficient

### Construct validity

All items had significantly stronger correlations with their own scales than with other scales, and no overlap was observed. Cronbach´s alpha coefficient indicated good internal consistency of the QLQ-LMC21, ranging from 0.84 to 0.89. The multitrait scaling analysis is presented in Table 4.

**Table 4:** Multitrait scaling analyses and reliability of the QLQ-LMC21 scales before and after treatment

	All patients at baseline (n = 235)			All patients at follow-up (n = 204)		
	Item correlation within scale*	Item correlation with other scale	Cronbach alpha	Item correlation within scale*	Item correlation with other scale	Cronbach alpha
QLQ-LMC21						
Nutritional problems	0.56 - 0.56	0.05 - 0.39	0.86	0.67 - 0.67	0.17 - 0.49	0.89
Fatigue	0.68 - 0.82	0.18 - 0.60	0.84	0.67 - 0.83	0.27 - 0.65	0.87
Pain	0.27 - 0.77	0.05 - 0.32	0.86	0.36 - 0.72	0.19 - 0.40	0.89
Emotional problems	0.39 - 0.73	0.15 - 0.60	0.85	0.44 - 0.70	0.21 - 0.65	0.88

*Corrected for overlap

### Concurrent validity

The results of the correlation analysis were similar for both preoperative and postoperative follow-up responses. Overall, the correlations were weak between the QLQ-C30 and QLQ-LMC21. Strong correlations were observed between the scales that were similar in the two instruments: fatigue, pain and emotional problems.

Stronger correlations (rho > 0.60) were also found between dissimilar scales, such as fatigue and emotional problems in the QLQ-LMC21 and global quality of life in the QLQ-C30. There was also a strong correlation (rho > 0.60) between physical functioning and role functioning in the QLQ-C30 and fatigue in the QLQ-LMC21 (Table 5).

**Table 5:** Correlations between scales in the QLQ-LMC21 at baseline and follow-up

QLQ-C30 scales	QLQ-LMC21 scales							
	Nutritional problems		Fatigue		Pain		Emotional problems	
	Baseline	Follow-up	Baseline	Follow-up	Baseline	Follow-up	Baseline	Follow-up
Physical function	-0.35*	-0.50*	-0.60*	-0.63*	-0.28*	-0.43*	-0.35*	-0.37*
Role function	-0.37*	-0.37*	-0.69*	-0.70*	-0.32*	-0.38*	-0.48*	-0.44*
Emotional function	-0.39*	-0.38*	-0.52*	-0.58*	-0.37*	-0.45*	-0.74*	-0.73*
Cognitive function	-0.41*	-0.35*	-0.50*	-0.42*	-0.30*	-0.37*	-0.41*	-0.43*
Social function	-0.31*	-0.38*	-0.56*	-0.62*	-0.27*	-0.38*	-0.50*	-0.54*
Global quality of life	-0.47*	-0.53*	-0.67*	-0.72*	-0.40*	-0.49*	-0.61*	-0.61*
Pain	0.23*	0.40*	0.34*	0.53*	0.56*	0.62*	0.37*	0.49*
Fatigue	0.45*	0.53*	0.71*	0.76*	0.41*	0.53*	0.50*	0.52*
Nausea and womiting	0.43*	0.39*	0.30*	0.38*	0.38*	0.40*	0.32*	0.26*

Values are correlation coeffients

*Correlation is significant at the 0.01 level (2-tailed)

## Discussion

The purpose of this multicentre study was to translate and evaluate the validity and reliability of the EORTC QLQ-LMC21 questionnaire module in a sample of 250 patients diagnosed with liver metastases from CRC who intended to undergo surgical intervention. The study was conducted at four Swedish university hospitals and had a high level of compliance. The majority of missing data (18% at follow-up) was from the question that assessed sexual function.

It is important to evaluate health-related quality of life for several reasons, including to individualize treatment and to investigate the effect of different treatments on patients'quality of life and not just on symptoms and survival. It is important to include the patient's perspective when developing and improving treatment methods. Patient-reported outcomes have been shown to increase person-centred care and may also have a prognostic value for survival, and they are a valuable tool for monitoring the patient's clinical status in clinical trials [12–18].

The results of this study indicate that the Swedish translation of the EORTC QLQ-LMC21 is a valid and reliable tool for measuring quality of life in patients with liver metastases from CRC treated with surgery. Importantly, responsiveness to change in health status over time was confirmed. This study also provides support for its use, together with the EORTC QLQ-C30, for clinical and epidemiological evaluations in a Swedish population.

The Swedish versions of the EORTC QLQ-C30 and EORTC QLQ-LMC21 were found to be sensitive to changes over time. The results are similar to those of the hepatectomy group in the original study except for item 4, taste problems, where the results in our study did not show any significant alteration indicating more symptoms [6].

Test–retest reliability aims to evaluate the degree to which an instrument can reproduce results over time in patients with stable conditions. The higher the ICC, the stronger the agreement between the measurements. An ICC > 0.70 is considered acceptable for group analyses and an ICC > 0.90 is recommended for assessment of individuals. An ICC of 0.70 to 0.90 is considered moderate or good reliability, and values ​​above 0.90 are considered high or excellent [11]. In this study, reproducibility was found to be good. Test–retest reliability has not been reported in any of the other studies validating the LMC21 except for the Polish version [6, 19, 20].

Construct validity examines how items relate to each other and to the scales. We found a weak correlation between questions 39 and 42 on the pain scale, and we also found a weak correlation between questions 47 and 50 on the emotional scale (Supplement 1). All items had significantly stronger correlations with their own scales than with other scales. The correlations exceeded the 0.40 criterion for convergent validity for all other scale correlations, which is largely consistent with the original study [6].

Concurrent validity indicates the amount of conformity between two different assessments. In this study, concurrent validity was consistent with the original study except for emotional problems and global quality of life [6]. Stronger correlations were found between global quality of life in the QLQ-C30 and fatigue and emotional problems in the QLQ-LMC21. There was also a strong correlation between physical function and role function in the QLQ-C30 and fatigue in the QLQ-LMC21. These findings confirm that these two questionnaires do not co-variate.

There are some limitations in our study. To evaluate the instrument’s ability to distinguish between subgroups of patients with different tumour burden in the liver, a known group comparison can be conducted [11]. In the original study, a known group comparison was made on a group of patients with liver metastases from CRC who received palliative treatment [6]. In our study, no known group comparison was made, which is a limitation. Logistical problems and restrictions during the pandemic prevented the implementation of this comparison in a palliative group. In this study, the mean age of the patients was 69 years, and there was a higher proportion of males than females and a higher proportion of stage 3 and 4 patients than stage 1 and 2 patients. This may reflect the nature of the disease, and similar results can be found in the literature [2, 6, 19–22]. Most of the patients had less than 10 years of education, they were married or cohabiting, and the majority were retired. For the most part, patients managed all normal activity with an ECOG status of 0, and the liver metastases were detected synchronously with the primary tumour. This patient cohort has similar sociodemographic characteristics to patients in previous studies that assessed the psychometric validity and reliability of the QLQ-LMC21 [6, 19, 20]. In this study, however, 30% of the patients had undergone liver surgery first.

In accordance with the EORTC Quality of Life Group's guidelines for developing quality-of-life instruments, a minimum of 10 patients per item is needed [23]. LMC21 consists of 21 items, so a total of 210 patients were calculated to be necessary for validation of the instrument. When the study was planned, a 20% dropout rate was calculated, so a total of 250 patients were included. Finally, 242 patients were analysed, and in the postoperative follow-up, only 15% of the patients were lost. This high response rate must be considered a strength.

The patients were recruited from four university hospitals in different parts of Sweden to obtain a representative sample of the population.

## Conclusions

In conclusion, despite these limitations, the results presented here indicate that the Swedish version of the EORTC QLQ-LMC21 is a valid and reliable questionnaire that has clinical validity as it is sensitive to changes in health before and after treatment. EORTC QLQ-LMC21 is recommended to use in combination with the EORTC QLQ-C30 in future clinical trials in Swedish populations of patients with liver metastases from CRC.

## Supplementary Information

Supplementary Material 1

## Data Availability

The data that support the findings of this study are not openly available due to reasons of sensitivity and are available from the corresponding author upon reasonable request. Data are located in controlled access data storage at Linköping University.

## Abbreviations

CRC: Colorectal cancer
EORTC: European Organization for Research and Treatment of Cancer
ICC: Intraclass correlation coefficient
