# Supplementary material for: Translation and validation of the Swedish version of the EORTC LMC-21, the disease-specific questionnaire for assessing health-related quality of life in patients with colorectal liver metastases
Source: BMC Gastroenterol. 2025 Apr 22;25:281. doi: 10.1186/s12876-025-03835-w (PMC12013156; doi:10.1186/s12876-025-03835-w)
Supplement: Supplementary file 1 — Supplementary Material 1 [file 12876_2025_3835_MOESM1_ESM.pdf]

EORTC QLQ – LMC21

Patients sometimes report that they have the following symptoms or problems. Please indicate the extent to which you have experienced these symptoms or problems during the past week. Please answer by circling the number that best applies to you.

**During the past week :**	**Not at All**	**A Little**	**Quite a Bit**	**Very Much**
31. Have you had trouble with eating?	1	2	3	4
32. Have you felt full up too quickly after beginning to eat?	1	2	3	4
33. Have you worried about losing weight?	1	2	3	4
34. Have you had problems with your sense of taste?	1	2	3	4
35. Have you had a dry mouth?	1	2	3	4
36. Have you had a sore mouth or tongue?	1	2	3	4
37. Have you been less active than you would like to be?	1	2	3	4
38. Have you had tingling hands or feet?	1	2	3	4
39. Have you had pain in your stomach area?	1	2	3	4
40. Have you had discomfort in your stomach area?	1	2	3	4
41. Have your skin or eyes been yellow (jaundiced)?	1	2	3	4
42. Have you had pain in your back?	1	2	3	4
43. Have you felt slowed down?	1	2	3	4
44. Have you felt lacking in energy?	1	2	3	4
45. Have you had trouble having social contact with friends?	1	2	3	4
46. Have you had trouble talking about your feelings to your family or friends?	1	2	3	4
47. Have you felt stressed?	1	2	3	4
48. Have you felt less able to enjoy yourself?	1	2	3	4
49. Have you worried about your health in the future?	1	2	3	4
50. Were you worried about your family in the future?	1	2	3	4
**During the past four weeks:**				
51. Has the disease or treatment affected your sex life (for the worse)?	1	2	3	4

Specimen

© QLQ-LMC21 Copyright 2002 EORTC Quality of life Group. All rights reserved
